# Cannabis use and subjective cognitive decline in Delaware: a sneak peek

**DOI:** 10.1192/j.eurpsy.2026.10449

**Published:** 2026-07-31

**Authors:** S. Gupta

**Affiliations:** Public and Allied Health sciences, Delaware State University, Dover, United States

## Abstract

**Introduction:**

Cannabis consumption has rapidly increased in Delaware due to the legalization of recreational use. There is limited research, however, investigating the links between cannabis use and cognition in older adults.

**Objectives:**

The objective of this study is to represent the first population-level analysis of cannabis use and subjective cognitive decline (SCD) in older adults in First State.

**Methods:**

Data were obtained from 4,282 Delaware adults in the 2023 Behavioral Risk Factor Surveillance System (BRFSS). Odds of SCD by cannabis use reason, frequency, and methods (e.g., smoke, eat, vaporize) were examined using multiple logistic regression and complex survey weighting methodology.

**Results:**

Compared to non-users, non-medical cannabis use was significantly associated with a 96% reduction in the odds of SCD (aOR=0.05, 95% CI=0.02-0.44, p<.01). Medical use (aOR=0.56, 95% CI=0.06-3.61, p=.46) and dual medical and non-medical use (aOR=0.40, 95% CI=0.03-2.92, p=.40) were also linked to reduced odds of SCD, though not significant. The frequency and method of cannabis consumption were not significantly associated with SCD.

**Conclusions:**

The reasons for cannabis use, excluding its frequency and method, are associated with SCD. Further research is necessary to explore the mechanisms that may contribute to the observed connections between non-medical cannabis use and decreased odds of SCD.

**Disclosure of Interest:**

None Declared

